# Supplementary material for: Content-rich biological network constructed by mining PubMed abstracts
Source: BMC Bioinformatics. 2004 Oct 8;5:147. doi: 10.1186/1471-2105-5-147 (PMC528731; doi:10.1186/1471-2105-5-147)
Supplement: Additional File 2 — The original results of the above study (non-essential files are deleted to keep the file size under the limit set by BMC bioinformatics). [file 1471-2105-5-147-S2.bz2 › chilibotAdditionalFile2/dip20/457ID10733106E10474/html/CCR3_CCL7.html]

 


 **CCR3** and **CCL7** 
  
Found 58 abstracts in PubMed, retrieved 20.  
 

 What does Google say? 
 PDF only 
| .edu only 

---

**Interactive relationship** (e.g. stimulation, inhibition, etc)

**Neutral relationship**- Therefore, interactions of eotaxin s, eotaxin CCL11, RANTES CCL5, and MCP 1 CCL2, MCP 2 CCL8, MCP 3  [ **CCL7** ]   **CCL7** , MCP 4 CCL13 with  **CCR3**  are responsible for the recruitment of basophils, eosinophils and mast cells.  Ref: 12009564 Mol Immunol, 2002
- Comparison of the structure of vMIP II with eotaxin 1, RANTES, and MCP 3  [ **CCL7** ]  suggests a unique mechanism for  **CCR3**  activation.  Ref: 11041848 Biochemistry, 2000
- Surface epitopes identified on RANTES to be involved in binding to  **CCR3**  are mimicked on the eotaxin 1 and MCP 3  [ **CCL7** ]  surface.  Ref: 11041848 Biochemistry, 2000
- The results presented here indicate that the structural basis for interaction with the chemokine receptor  **CCR3**  by vMIP II is different from that for the physiological agonists eotaxin 1, RANTES, and MCP 3  [ **CCL7** ] .  Ref: 11041848 Biochemistry, 2000
- **CCR3**  is known to be a ligand for CC chemokines such as RANTES, eotaxin and monocyte chemotactic protein 3  [ **CCL7** ]  MCP 3  [ **CCL7** ] .  Ref: 11240271 J Dermatol Sci, 2001
- I TAC, Mig, and IP10 compete for the binding of eotaxin to  **CCR3**  bearing cells and inhibit migration and Ca 2 changes induced in such cells by stimulation with eotaxin, eotaxin 2, MCP 2 monocyte chemottractant protein 2, MCP 3  [ **CCL7** ] , MCP 4, and RANTES regulated on activation normal T cell expressed and secreted.  Ref: 11110785 J Biol Chem, 2001

**Non-interactive relationship** (e.g. studied together, co-existance, homology, etc.)

- The chemokine receptor  **CCR3**  and ligands monocyte chemoattractant protein MCP 3  [ **CCL7** ] , MCP 4, regulated upon activation normal T cell expressed and secreted RANTES and eotaxins I and II are of particular relevance for the recruitment and activation of eosinophils.  Ref: 11340325 Int Arch Allergy Immunol, 2001
- Our findings point to a role for C C chemokine receptor 2 CCR2 and  **CCR3**  agonists such as monocyte chemotactic proteins MCPs 1 CCL  **CCL7**  and 5 CCL12 as important participants that are subject to regulation by Th2 cytokines interleukin IL 4 and IL 13.  Ref: 12102713 Parasite Immunol, 2002
- O 3 increased lung protein levels of CXCL10,  **CCL7** , and  **CCR3**  CCL7R.  Ref: 11777981 J Immunol, 2002
- We investigated tissue samples and tumor cell suspensions of patients with CD30 CTCL and CD30 CTCL for expression of the chemokine receptors  **CCR3** , CCR4, and CCR8 and the  **CCR3**  ligands eotaxin CCL11, monocyte chemoattractant protein 3  [ **CCL7** ]   **CCL7** , and RANTES CCL5.  Ref: 12393570 Blood, 2003
- These ligands include the  **CCR3**  selective chemokines eotaxin and eotaxin 2 and the more promiscuous CC chemokines, MCP 4, MCP 3  [ **CCL7** ] , MCP 2 and RANTES.  Ref: 11306952 Int Arch Allergy Immunol< MedlineTA>Int Arch Allergy Immunol, 2003
- We have compared the surface topology and electrostatic potential of vMIP II to those of eotaxin 1, RANTES, and MCP 3  [ **CCL7** ] , three  **CCR3**  physiological agonists with known three dimensional structures.  Ref: 11041848 Biochemistry, 2000
- In contrast, responses to MCP 3  [ **CCL7** ]  were blocked only in the presence of both mAbs, but not after pretreatment with either anti CCR2 or anti  **CCR3**  mAb alone.  Ref: 11120855 J Immunol, 2000
- OBJECTIVE AND DESIGN The gene expression profile of  **CCR3**  ligands, eotaxin, RANTES, and monocyte chemoattractant protein 3  [ **CCL7** ]  MCP 3  [ **CCL7** ] , was examined in normal and inflamed guinea pig lungs.  Ref: 11822789 Inflamm Res, 2001
